# Towards a ‘manifesto’ for super‐recognizer research

**DOI:** 10.1111/bjop.12411

**Published:** 2019-06-23

**Authors:** Meike Ramon, Anna K. Bobak, David White

**Affiliations:** ^1^ Applied Face Cognition Lab University of Fribourg Switzerland; ^2^ Department of Psychology Faculty of Natural Sciences University of Stirling UK; ^3^ School of Psychology UNSW Sydney New South Wales Australia

**Keywords:** Super‐recognizers, face processing, face recognition, face identification, face matching, open science, replicability

## Abstract

This article provides a response to five excellent commentaries on our article ‘Super‐recognizers: From the lab to the world and back again’. Specifically, the response summarizes commonalities between these commentaries. Based on this consensus, we propose a flexible framework for the assessment of superior face recognition and outline guiding principles to advance future work in the field.

## Bridging the gap between the laboratory and the world

Our target article was intended to encourage greater synergy between face recognition researchers and practitioners to develop knowledge of super‐recognizers (SRs) in the future. This is critical because the application of knowledge in this area has preceded development of a solid theoretical knowledge base. Collaboration between practitioners and academics is vital to redress this and in order to implement and evaluate procedures to meet current and future real‐world demands (Ramon, Bobak, & White, 2019).

Twelve respected researchers took the time to respond thoughtfully to our article, and to extend the ideas we put forward. Together, these responses reflect the vast interest in this topic over recent years and the positive steps that are already underway to address the gap between the laboratory and the world (e.g., in test development; Robertson & Bindemann, 2019; Bate, Portch, Mestry, & Bennetts, 2019; Devue, 2019), including the emergence of collaborative groups comprising academics and experienced face identification practitioners (Moreton, Pike, & Havard, 2019).

Substantial agreement emerged on the following key points. First, there is broad consensus that caution should be exercised in deploying SRs to perform real‐world tasks, given the limited level of scientific understanding, and paucity of data on validity and reliability of selection tasks for diverse real‐world deployments. Second, others shared our specific concerns that the quasi‐scientific claims made by commercial organizations and in popular media are likely to exacerbate this problem (Bate *et al*., 2019; Robertson & Bindemann, 2019). Third, there is an unequivocal agreement that closer collaboration between practitioners and academics is required to establish and ensure rigorous and reliable testing practices.

While it is not possible to give all points raised in the commentaries the full consideration that they deserve here, our hope is that these aspects will be expanded upon in future work. In the following sections, we attempt to draw out some key issues and areas of overlap, in an attempt to map out a potential direction for this research effort in the future. Our aim is to work towards a framework and set of common goals whilst preserving the healthy, diverse approach that has characterized research in this area.

## The need for a flexible and efficient framework to assess face processing abilities

The opinions voiced in this scientific exchange indicate that aiming for a standard battery of specific tests to identify SRs would be a suboptimal approach. The main reason is that any such potential agreement is insufficiently flexible to accommodate the continuously changing demands of real‐world challenges that practitioners are confronted with. Therefore, the more realistic and pragmatic approach is to conceptualize a *framework* for assessing discrete and distinguishable cognitive (sub)processes (Bate *et al*., 2019; Devue, 2019; Ramon *et al*., 2019), as well as clearly defined tasks of interest (Devue, 2019; Moreton *et al*., 2019; Ramon *et al*., 2019). Ideally, this framework would be adopted by researchers and practitioners from a range of disciplines interested in identifying individuals with superior processing abilities – be it for deployment or fundamental research purposes.

On a practical level, this framework should consider the *nature* of superior processing that is aimed to be identified (*face* vs. *person* identity processing? Bate *et al*., 2019), as well as the specific roles which to‐be‐selected individuals are expected to perform (e.g., passport control or crowd search? Moreton *et al*., 2019; Ramon *et al*., 2019). The framework should also incorporate guidelines to ensure selection of experimental procedures most suitable for assessing specific roles and identifying abilities that are critical in that specific operational context. These procedures for assessment must (1) incorporate multiple tests *and* measures within these tests (e.g., accuracy and reaction time (see Stacchi, Huguenin‐Elie, Caldara, & Ramon, 2019), and (2) ensure that individuals are identified *accurately* and *reliably* as SRs (Bate *et al*., 2019; Young & Noyes, 2019; cf. Wilmer *et al*., 2012). That is, all adopted procedures require sufficient psychometric calibration to meet the criterion of valid and reliable diagnostic sensitivity (see also Bate *et al*., 2018; Bobak, Pampoulov, & Bate, 2016; Stacchi, *et al*., 2019).

The practices developed under this framework will ultimately serve to characterize the boundaries and biases associated with superior ability, in order to best match individuals with the highly varied roles and contexts that characterize real‐world tasks (Bate *et al*., 2019; Devue, 2019). To the extent that the tests are reliable, this approach can also provide theoretical insights into associated and dissociated abilities within the person perception system (Young & Noyes, 2019; cf. Bate *et al*., 2018). Developing such a framework in the years ahead will inevitably rely on scientists and practitioners’ willingness to communicate and share knowledge and practices.

## Setting knowledge free

Researchers and practitioners may, in principle, agree on conceptual aspects and specific working frameworks, which, however, may not always translate into working practices. To ensure progress in this field, we propose the following guidelines, with the common goal of making the work more transparent and replicable. These recommendations to academics, practitioners and other end‐users, and the government are neither exhaustive, nor provided in order of importance, but are offered as a guide for future practice in theoretical and applied SR research.

Firstly, the research into superior face processing abilities should proceed via a long‐term collective goal of *expanding the body of knowledge* and *improving practices*. We echo the view that if individuals with exceptional face or person recognition skills are, indeed, superior to typical perceivers, they should be deployed in professions where their abilities may help make societies fairer and safer (cf, Young & Noyes, 2019). This should be in service of improving scientific understanding and lead to the betterment of society by achieving measurable practical gains in, for example, policing, rather than serving private interests. Practitioners and scientists have a shared power to achieve such goals and, as pointed out by our peers, should work together to avoid filling this vacuum by private enterprise whose prerogative is a financial gain (see also Robertson & Bindemann, 2019).

This endeavour can be accomplished through *close collaboration between scientists and practitioners* (see also Moreton *et al*., 2019). Working groups and consortiums with academics and practitioners and other end‐users foster collaborations which are at the core of progress of this field. Such knowledge exchange is critical for developing of valid and reliable tasks that reflect cognitive processes employed in real‐world assignments (Devue, 2019; Young & Noyes, 2019). This, in turn, would allow researchers to identify the best people for various roles ‘in the wild’. Additionally, multilaboratory collaborations involving large groups of individuals with superior processing skills can help identify patterns and provide a more detailed understanding of individual differences, which is currently lacking.

Such projects must not be constrained by the boundaries of specific laboratories or research collaborations, but *benefit from sharing of knowledge, procedures, and data*. Given the paucity of SRs, it is pertinent that detailed information concerning procedures is accessible for researchers outside a specific laboratory. With adherence to local data protection laws and practices, individual cases and procedures should be scrutinized by researchers and practitioners worldwide. Such collaborative *work should always be open to critique*; the field of superior face processing should represent no exception. Academic peer review plays an important role in controlling the quality of science and can provide an objective means of quality control in non‐academic settings.

Finally, we acknowledge that full transparency in the context of collaborations with non‐academic partners is not always feasible. For example, some research contracts in police and security agencies often hold complete discretion over the publication and dissemination of results. This is a significant challenge for researchers that aim to improve knowledge of SRs through the reverse‐translational approach we argue for in our target article. Nevertheless, scientists have a shared responsibility to act as advocates for ‘setting knowledge free’: our actions can help ensure that (potential) research partners appreciate that the ‘real world’ is not the end‐point of the knowledge cycle. We hope that the framework outlined in our initial proposal, as well as our colleagues’ independent calls for transparency, can help researchers, practitioners, and other end‐users to make this approach a ‘gold standard’ in the years ahead.

The science of SRs will have a substantial impact on the way that facial identity information is processed in organizations of the future. Our research decisions and the way we communicate our findings will all have tangible effects on a variety of critical legal, quasi‐legal, and security processes. For now, this collection of articles appears to be a useful starting point for academics and practitioners to work towards the common goals that have been identified here.
